# Guided Internet-Based Parent Training for Challenging Behavior in Children With Fetal Alcohol Spectrum Disorder (Strongest Families FASD): Study Protocol for a Randomized Controlled Trial

**DOI:** 10.2196/resprot.4723

**Published:** 2015-10-13

**Authors:** Karen Turner, James N Reynolds, Patrick McGrath, Patricia Lingley-Pottie, Anna Huguet, Amy Hewitt, Courtney Green, Lori Wozney, Christopher Mushquash, Nazeem Muhajarine, Andre Sourander, Heather Caughey, Jessica Roane

**Affiliations:** ^1^ IWK Health Centre Halifax, NS Canada; ^2^ Centre for Neuroscience Studies Queen's University Kingston, ON Canada; ^3^ Department of Biomedical and Molecular Sciences Queen's University Kingston, ON Canada; ^4^ Departments of Psychiatry and Pediatrics Faculty of Science Dalhousie University Halifax, NS Canada; ^5^ Department of Community Health and Epidemiology Dalhousie University Halifax, NS Canada; ^6^ Dalhousie University Halifax, NS Canada; ^7^ Department of Psychology and Northern Ontario School of Medicine Lakehead University Thunder Bay, ON Canada; ^8^ Department of Community Health and Epidemiology University of Saskatchewan Saskatoon, SK Canada; ^9^ Research Centre for Child Psychiatry University of Turku Turku Finland; ^10^ Public Health Agency of Canada Ottawa, ON Canada

**Keywords:** fetal alcohol spectrum disorder, neurobehavioral disorder, prenatal alcohol exposure, disruptive behavior, children, Strongest Families, parenting, randomized controlled trial, eHealth, Web-based intervention

## Abstract

**Background:**

Fetal alcohol spectrum disorder (FASD) is a term used to encompass the full range of neurobehavioral and cognitive dysfunction that may occur as a consequence of prenatal alcohol exposure. There is relatively little research on intervention strategies that specifically target the behavioral problems of children with FASD. Availability and access to services are barriers to timely and effective care for families. The Strongest Families FASD intervention was recently adapted from the Strongest Families “Parenting the Active Child” program to include FASD-specific content delivered via an Internet-based application in conjunction with 11 telephone coaching sessions.

**Objective:**

Our objectives are to (1) evaluate the effectiveness of Strongest Families FASD in reducing externalizing problems (primary outcome), internalizing problems, and parent distress (secondary outcomes) in children aged between 4 and 12 years diagnosed with FASD when compared to a control group with access to a static resource Web page; (2) evaluate the effectiveness of Strongest Families FASD in improving social competence (secondary outcome) in school-aged children aged between 6 and 12 diagnosed with FASD when compared with an online psychoeducation control; and (3) explore parental satisfaction with the Strongest Families FASD online parenting program.

**Methods:**

Parents and caregivers (N=200) of children diagnosed with FASD who have significant behavioral challenges, ages 4-12, are being recruited into a 2-arm randomized trial. The trial is designed to evaluate the effectiveness of the Web-based Strongest Families FASD parenting intervention on child behavior and caregiver distress, compared to a control group receiving access to a static resource Web page (ie, a list of FASD-specific websites, readings, videos, and organizations).

**Results:**

The primary outcome will be externalizing problems measured by the Child Behavior Checklist (CBCL). Secondary outcomes include (1) internalizing problems and (2) social competence, both measured by the CBCL; and (3) parental distress measured by the Depression Anxiety Stress Scale-21. The Client Satisfaction Questionnaire-8 (CSQ-8) and the Satisfaction Survey are completed by the intervention group at the end of session 11. Results will be reported using the standards set out in the Consolidated Standards of Reporting Trials (CONSORT) Statement.

**Conclusions:**

It is hypothesized that the Strongest Families FASD intervention group will improve child behavior and parental distress. Caregiver satisfaction is anticipated to be positive. Advancing evidence on the effectiveness and acceptance of distance services can inform policy and adoption of eHealth programs.

**ClinicalTrial:**

ClinicalTrials.gov NCT02210455; https://clinicaltrials.gov/ct2/show/NCT02210455 (Archived by WebCite at http://www.webcitation.org/6bbW5BSsT)

##  Introduction

Prenatal alcohol (ethanol) exposure is the leading known cause of developmental disability in Canada and is the most prevalent preventable cause of congenital neurobehavioral dysfunction in the Western world [1]. Despite attempts to increase public awareness of the risks associated with drinking during pregnancy, a report from the Public Health Agency of Canada indicates that a significant proportion (10.5%) of pregnancies in Canada are alcohol exposed [2]. The term fetal alcohol syndrome (FAS) was introduced over 40 years ago [3-5] as a diagnosis for children who exhibit the triad of central nervous system (CNS) dysfunction, growth deficiency, and characteristic craniofacial dysmorphology resulting from maternal consumption of alcohol during pregnancy. Of these features, it is the CNS injury that is most debilitating and can manifest as intellectual, neurological, and behavioral abnormalities. More recently, the term fetal alcohol spectrum disorder (FASD) was established to encompass the full spectrum of teratogenic effects induced by ethanol [6]. FAS is believed to occur in approximately 1 to 3 per 1000 live births in North America, and it is estimated that FASD may occur as frequently as 1 in 100 live births [7]. Moreover, recent epidemiological studies suggest prevalence rates as high as 2%-5% [8]. In addition, the total adjusted annual cost associated with FASD in Canada was estimated at US $5.3 billion [9]. The substantial personal impacts of FASD to Canadian families coupled with the economic costs illustrate the need for evidence-based prevention and early intervention.

A key goal of the Public Health Agency of Canada’s FASD initiative is to improve the outcomes for people living with FASD. The agency does this by developing practical resources and tools for professionals that build understanding and health professional capacity. These resources complement the substantial work undertaken by provincial and territorial governments to increase diagnostic capacity for FASD [7]. Despite these improvements, there remain challenges with the initial identification of individuals who may be potentially affected by FASD, as well as significant logistical issues, including access to diagnosis in rural and isolated communities.

Once diagnosed, the need for services and supports for children and their families remains largely unmet [10]. Parents and caregivers (henceforth referred to as “parents”) of children with FASD are often confronted with significant behavioral challenges without adequate resources and information to help them manage these symptoms [11]. Despite a variety of psychosocial interventions aimed at supporting individuals with neurobehavioral disorders, relatively little research exists that is specifically aimed at improving the behavioral challenges associated with FASD [12]. Problems with service delivery are further compounded in smaller centers and in rural and isolated communities where access to specialized support programs is extremely limited. Even when services are available, significant barriers—such as the costs associated with traveling to clinics for repeated consultations and time spent away from work—make it difficult for families to benefit from them. Additionally, frequent turnover of program personnel often leads to uneven quality and consistency of program delivery. Carefully designed and implemented alternative service delivery models have the potential to meet the needs of children and families with FASD where traditional models have shown difficulty.

The Strongest Families FASD Parenting Program is adapted from the evidence-based Strongest Families “Parenting the Active Child Program” [13-15], which was designed to address many of these issues by providing distance services to families with children exhibiting behavior problems. Program content from the Strongest Families Institute (SFI), a not-for-profit that provides a number of distance-based psychosocial interventions to children and families, was revised to include information specific to FASD, relevant examples, and modification of some curriculum skills. The content is housed within Intelligent Research and Intervention Software (IRIS) [16], a secure Web-based platform that supports interactive and persuasive system design features (eg, personalization, reminders). Via IRIS, parents work online through a progressive skill-based curriculum that includes interactive exercises, instructional videos, and audio clips. Coaches are able to log in to IRIS to see the activities the parent has completed, facilitating their role of supporting parents throughout the program. Telephone coaching sessions are scheduled at convenient times for the parent, thereby removing barriers to care and providing services in the comfort and privacy of their own homes. Participants receive study emails via a secure inbox within IRIS.

### Objectives

Our first objective is to evaluate the effectiveness of the Strongest Families FASD program in reducing externalizing problems (primary outcome), internalizing problems, and parent distress (secondary outcomes) in children aged between 4 and 12 years old diagnosed with FASD as compared to a control group with access to a static resource Web page.

We hypothesize that the effects of the Strongest Families FASD intervention will be to reduce the child’s externalizing and internalizing problems (as measured by the Child Behavior Checklist (CBCL) externalizing and internalizing scales) and reduce parental distress (as measured by the Depression Anxiety and Stress Scale-Short Form, DASS-21), compared to the control group.

Our second objective is to evaluate the effectiveness of the Strongest Families FASD program in improving social competence (secondary outcome) in school-aged children between 6 and 12 years of age diagnosed with FASD as compared to an online psychoeducation control.

We hypothesize that the effects of the Strongest Families FASD intervention will be to improve the child’s social competence as measured by CBCL Social Competence Scale compared to that for the control group.

Our final objective is to explore parental satisfaction with the Strongest Families FASD online parenting program.

##  Methods

### Study Design

**Figure 1:**
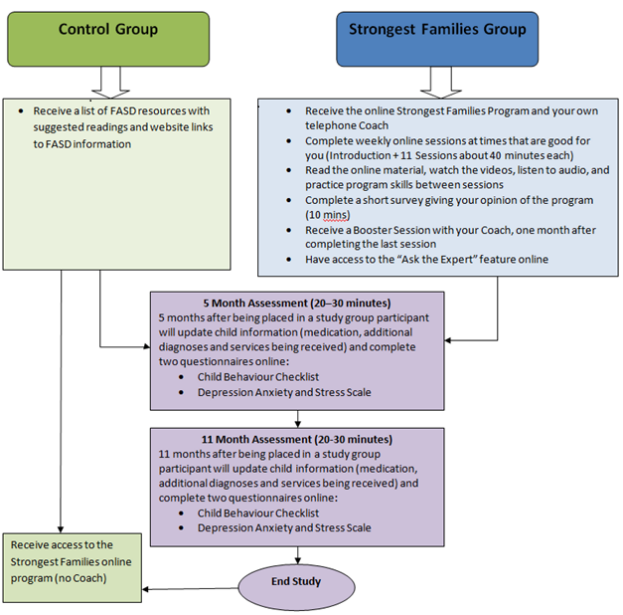
Study flow.

This study is a 2-arm randomized controlled trial comparing participants assigned to receive either the Strongest Families FASD Web-based parent-training program (SF Intervention Group) or a static resource Web page (Control Group). Neither group will be restricted from accessing additional programs or services during study participation. The Consolidated Standards of Reporting Trials (CONSORT) recommendations [17] are used to guide the methodology. Details of the study design are illustrated in Figure 1. The study has been approved by the Queen’s University Health Sciences & Affiliated Teaching Hospitals and IWK Health Centre Research Ethics Boards. The primary and secondary outcome measures will be administered to all participants at baseline (pre-randomization), 5 months, and 11 months post-randomization. The Client Satisfaction Questionnaire (CSQ-8) and Strongest Families Program Satisfaction Questionnaire: FASD Version will be administered to the SF Intervention Group upon completion of the program. Study participation ends for participants in both study groups after completing the 11-Month Follow-up Assessment.

### Measures

#### Child Behavior Checklist

The CBCL is a standardized questionnaire that assesses a child’s adaptive functioning and behavior problems by asking parents to rate the frequency with which their child displays each of a series of behaviors. Most items are measured on a 3-point Likert-scale, where 0 means “Not True (as far as you know)” and 2 means “Very True or Often True.” Scores are then summed across a number of different subscales and compared to a normative sample to provide a *t*-score for each subscale indicating if the child’s behavior is within “normal” limits, “borderline clinical,” or “clinical.” It is demonstrably sensitive to the effects of parent training programs [18,19]. We will use 2 parent-report versions of the CBCL—the preschool version (CBCL/1½-5) [20] and the school-age version (CBCL/6-18) [21]—to accommodate the age range of the sample. The CBCL requires 15-20 minutes to complete.

##### CBCL/1½-5

The CBCL/1½-5 consists of 7 syndrome scales (eg, Emotionally Reactive, Anxious/Depressed, Somatic Complaints, Withdrawn, Sleep Problems, Attention Problems, and Aggressive Behavior). Attention Problems and Aggressive Behavior items are grouped into an Externalizing Factor. The remaining scales, with the exception of Sleep Problems, are grouped into an Internalizing Factor. Overall test-retest reliability over 2 test periods averaging 8 days is reported as *r=* 0.85. Reliability for the 24-item Externalizing and 36-item Internalizing Scales is reported at alpha=.87 and alpha=.90, respectively. Internal consistency is reported for the Externalizing Scale at alpha=.92 and for the Internalizing scale at alpha=.89 [20].

##### CBCL/6-18

In addition to asking parents to rate frequency of their child’s behaviors, the CBCL/6-18 asks parents to provide information about their child’s participation in academic, social, and extracurricular activities. The CBCL/6-18 consists of 8 syndrome scales (eg, Anxious/Depressed, Withdrawn/Depressed, Somatic Complaints, Social Problems, Thought Problems, Attention Problems, Rule-Breaking Behavior, and Aggressive Behavior), which group into 2 higher order factors: Internalizing and Externalizing behaviors. This version of the CBCL also provides competence scales for activities, social relationships, and school performance and a total competence score. Overall 8-day test-retest reliability was *r*=0.92. Reliability for the 35-item Externalizing and 32-item Internalizing scales are alpha=.92 and alpha=.91, respectively. Internal consistency for the Externalizing and Internalizing scales are alpha=.94 and alpha=.90, respectively [21].

#### Depression Anxiety and Stress Scale-Short Form

The DASS-21 [22] will be used to evaluate parent distress. The DASS-21 is a 21-item self-report measure that asks participants to indicate the extent to which they have experienced a series of mental health symptoms in the past week, ranging from 0 (did not apply to me at all) to 3 (applied to me very much or most of the time). Items belong to 1 of 3 subscales (eg, depression, anxiety, and stress) that can be combined into a composite measure of general distress [23]. The DASS-21 demonstrates strong internal consistency with alpha values at .84 for anxiety, .90 for stress and .91 for depression [22]. The DASS-21 has proven sensitive to the effects of parent-focused interventions [24] and requires 5-10 minutes to complete.

#### Client Satisfaction Questionnaire

The CSQ-8 has been widely used in primary care and mental health treatment to measure patient/client satisfaction with services received [25]. The CSQ-8 consists of 8 items asking patients/clients to rate the services on a 4-point Likert scale. An overall score for the CQS-8 is produced by summing all item responses. Scores range from 8 to 32, with higher values indicating higher satisfaction. Internal consistency for the CSQ-8 is reported with alphas ranging from .83 to .93 [26]. Participants (SF Intervention Group only) will be asked to rate the quality of service they received as part of the Strongest Families FASD Program.

#### Strongest Families Program Satisfaction Questionnaire: FASD Version

The in-house developed Strongest Families Program Satisfaction Questionnaire: FASD Version asks participants to rate their agreement with statements about the Strongest Families Program specific to coaching, program components (eg, written materials, videos, etc), and the website on a 5-point scale (strongly agree to strongly disagree). A lower score indicates higher satisfaction. The psychometric properties of this tool have not been tested.

### Sample

A sample of 200 parents of children with FASD, aged 4-12 years, will be recruited into the study. Our sample size estimate was based on the minimal clinically important difference in change in outcomes from 0 to 5 months. We have expressed this effect size as a moderate (d=0.50; ie, one half a standard deviation) difference in reduction on CBCL externalizing score for intervention group compared to control. Setting our Type I error rate (alpha) at 0.05. Thus, we require 85 participants in each group for a power of 0.90 and a total sample size of 170 (we will recruit 200 to account for losses). It is reasonable to expect this effect given the larger effects seen in children with oppositional defiant disorder and attention-deficit hyperactivity disorder [12]. The age range for the sample was chosen because interventions provided at this stage can help to prevent the development of secondary problems and because parent-training methods have been most highly developed for this age group [27]. The target sample size is attainable based on the strong relationships we have with FASD diagnostic clinics and FASD support groups across Canada.

### Recruitment

The recruitment strategy for this study will be broadly based across Canada. Advertisement materials will include posters, brochures, Web ads, promotional videos, and social media postings. Social media posts (eg, Facebook, Twitter, LinkedIn) will be used to increase study visibility and reach our target sample. Text for social media posts will change frequently to maintain interest in the study, encourage “sharing,” and extend recruitment reach. Also, our collaborators at Queen’s University have informed former study participants, who agreed to be contacted for future research opportunities, about this study. Partnerships with FASD clinics and support programs across Canada have been established and maintained with study progress updates. The study team promotes the study through conference presentations and exhibits. All interested individuals are directed to a study recruitment website (myStudies) to receive study information, screen for eligibility, and complete online consent (if eligible). The use of myStudies as a medium for screening and consent has been approved by the IWK Research Ethics Board.

#### Eligibility Screening (Phase 1)

Eligibility screening is a 2-step process that involves a preliminary online screening and then a more comprehensive screening process.

Phase 1 preliminary online screening is conducted on the myStudies website, where potential participants will be invited to complete screening questions for eligibility. Individuals may request (via the website) contact with study staff if they would like to speak to someone about the study. Primary caregivers who are not legal guardians (eg, foster parents, relatives) of the child to whom the study pertains will be required to obtain authorization from the child’s legal guardian before taking part in the study. Answers to the screening questions will be automatically assessed in myStudies using predefined acceptance criteria and individuals will immediately receive an on-screen message stating whether they are eligible or ineligible to continue to the next step. Individuals who are eligible at screening will be invited to proceed to an online consent process, also hosted on the myStudies site. Individuals who are ineligible will be informed that they do not meet the study requirements, thanked for their interest in the study, and encouraged to take part in future studies.

Parents must meet all of the following criteria to be eligible to participate in the study:

1. Have a child between 4-12 years of age with a diagnosis under the umbrella term Fetal Alcohol Spectrum Disorder (as reported by parents) who has been experiencing behavioral problems (as defined by the parent) for at least 6 months prior to study screening;
2. Have been the primary caregiver for a minimum of 6 months prior to entry into the study;
3. Have a reasonable expectation of being be the primary caregiver for at least 6 months after study enrolment;
4. Have the ability to read, write, and understand English;
5. Have access to a telephone;
6. Have access to a computer connected to the Internet; and
7. Live in Canada.

Endorsement of any of the following criteria will exclude parents from taking part in the study:

1. Child is not able to speak in full sentences or understand everyday language and instructions from parent;
2. Parent or child has been diagnosed with psychosis;
3. Child has been diagnosed with schizophrenia, bipolar disorder, or major depression;
4. Child has been identified as putting others at risk of serious harm (ie, requiring medical attention);
5. Parent has taken part in Triple P, COPE, or Incredible Years parenting program within 6 months of completing the study screening; or
6. Parent has previously taken part in a Strongest Families Parenting Program.

The nature of the intervention program necessitates exclusion criterion 1 to ensure that any issues with developmental/cognitive functioning will not impact the child’s ability to respond to basic parental instructions related to program skills. Exclusion criteria 2, 3, and 4 are designed to safeguard against the risk of enrolling individuals with potentially complex needs for which the program or study is not appropriate. Criteria 5 and 6 pertain to the parents’ experience with programs that teach similar skills.

#### Eligibility Screening (Phase 2)

Phase 2 screening involves completion of the CBCL baseline measure to determine eligibility for randomization. DASS-21 scores will not impact eligibility. All participants who complete the baseline assessment receive a US $25 gift card by mail or email (their choice).

The following criteria must be met in order for participants to be randomized into a study group:

1. CBCL Externalizing t-Score ≥64 (clinical range);
2. Parental report of a formal diagnosis of one of the following disorders captured under the umbrella term FASD: Fetal Alcohol Syndrome (FAS); partial fetal alcohol syndrome; alcohol related neurodevelopmental disorder; static encephalopathy (alcohol exposed); neurobehavioral disorder (alcohol exposed); and other (To determine possible valid diagnoses not included on our predefined list, parents may report an “other” diagnosis that they believe falls under the umbrella term FASD. These diagnoses will be confirmed by the co-principal investigator on a case-by-case basis and will be accepted if the diagnosis identified (a) has been provided by a recognized FASD diagnostic clinic and (b) conforms to one of the recognized diagnostic schemes used in the assessment of children suspected of having an FASD.);
3. Meets the following criteria for behavior suggestive of FASD adapted from the Neurobehavioral Screening Tool (NST) [28,29]: (a) For children age 6-12 (CBCL/6-18), participants must endorse 7 out of 8 of the items listed in Table 1; or (b) For children age 4-5 (CBCL/1½-5), participants must endorse 3 out of 5 of the items listed in Table 1 with at least 2 endorsements from the items marked with an asterisk.
4. No suicide attempts within the previous 6 months for children 6-12 years of age (screened for using the CBCL/6-18 only); and
5. No current risk of suicide attempts for children 6-12 years of age (screened for using the CBCL/6-18 only).

**Table 1:** Parent endorsements required to meet clinical eligibility criteria.

CBCL Item	CBCL/1½-5	CBCL/6-18
Acts young for age	✓	✓
Can’t concentrate or poor attention*	✓	✓
Can’t sit still, restless, hyperactive*	✓	✓
Disobedient at home*	✓	✓
Doesn’t show guilt after misbehaving	✓	✓
Argues a lot		✓
Impulsive or acts without thinking		✓
Lying or cheating		✓

Endorsement of CBCL/6-18 item 18 (deliberately harms self or attempts suicide) will generate automatic probes for details within IRIS. Endorsement of current suicide risk for a child who is not being monitored by a health care professional will prompt an automatic onscreen recommendation to seek professional help. This recommendation will be re-iterated to the participant (parent) in the Assessments Results Letter and followed up with a phone call from study staff.

### Randomization and Allocation

Random allocation to the intervention or control group in 1:1 ratio was established independently by an external researcher using a permuted blocked randomization procedure. The external researcher concealed the group placements in sequentially numbered, sealed double envelopes to maintain blinding of study staff. Envelopes are opened sequentially by the coordinator (or delegate) and only after the enrolled participants complete the baseline assessments and are deemed eligible.

### Intervention Group

The online Strongest Families FASD program is provided to the parent with weekly telephone coaching sessions; there is no contact with the child. The program consists of evidence-based parenting strategies provided in 11 online sessions using easy-to-read text, demonstrative video and audio clips, interactive questionnaires, and practice exercises. Participants enter information (eg, child’s name and specific behavior problems) into IRIS to customize the program content. The weekly coaching sessions facilitate skill acquisition and successful implementation using problem-solving and role-playing techniques. Supplementary program materials (eg, Reward Chart, a Daily Strengths Chart, a Visual Schedule Template, Tryout Pages, and Tips for Teachers Information Sheet) are sent to participants via mail. These materials are provided in physical format to enable parents to post the materials in a convenient place in the home and to facilitate communication between home and school or day care. A booster telephone coaching session is offered 1 month after completion of the program to check in with the parent to review consistent use of the skills, prevent or deal with setbacks, and provide support and encouragement, if needed. An experienced coach supervisor will conduct weekly case review with coaches to problem-solve and to review quality assurance and adherence to protocols.

The effectiveness of the principal components of the intervention has been supported in previous studies [13-15] and are currently being used by the SFI Service Program. The content was revised to tailor the intervention to the needs and challenges specific to families affected by FASD. Changes were informed by data collected in a series of telephone interviews with families and clinicians who have personal and professional interest and expertise in the field [30].

An “Ask the Experts” message board feature within IRIS allows parents to receive answers to individual questions from 1 of 2 designated experts with extensive clinical and research experience within the FASD population. A question deemed by the Principal Investigator (or delegate) to be of potential interest to the larger group will be de-identified and posted to the message board with a response. The author of that question will be notified by email and directed to the message board to view the response. Questions that are not appropriate or deemed not of potential interest for the larger group will be answered individually via email.

Participants receive progress reports midway through and at the end of the program, summarizing parent ratings of their child’s improvement across sessions since the beginning of the program. The parent submits ratings at the beginning of the next online session (eg, parent rates Session 1 at the beginning of Session 2) so that they have time to practice the previous skill and evaluate their child’s improvement.

Participants who do not maintain regular contact with their coaches will be sent a Re-Contact Letter via email after three consecutive missed phone calls followed by 5 days without response to an email from the coach. The file will be placed on temporary hold until the participant re-establishes contact or closed at the end of the projected study period. Automatic email reminders will be sent to the participant approximately every 6 weeks, inviting them to resume the program or to complete assessments.

### Control Group

The Control Group will receive access to a static (ie, noninteractive) FASD resource Web page within IRIS that provides a list of recommended book titles, videos, websites, and organizations.

Participants in the Control Group receive a Mid-Study Progress Message approximately 10 weeks after randomization (coinciding with the SF Intervention Group’s mid-program progress report). The message thanks participants for taking part in the study, encourages them to visit the static resource page, and reminds them of the 5-Month Follow-up Assessment.

Participants in the Control Group will gain access to the Strongest Families FASD program (without coaching) after their study participation is complete. The program is designed to be self-directed and contains built-in tutorials to help users navigate the website, though technical support is available. The Strongest Families program will be accessible until 6 months after the last Control Group participant receives access to the website.

### Follow Up Assessments

Participants will complete follow-up assessments at 5 and 11 months post-randomization. Participants in both study groups will receive an email message at 145 days (for the 5-month assessment) and 325 days (for the 11-month assessment) prompting them to complete the appropriate follow-up assessments online within IRIS. The assessments will take approximately 20-30 minutes in total to complete. Participants will be asked to complete the DASS-21 and the CBCL and to update some demographic information (eg, child’s medications, any new diagnoses). Up to 3 email reminders will be sent and a courtesy phone call reminder will be made for incomplete assessments approaching their expiry date. For each completed follow-up assessment, participants will receive a US $25 gift card by mail or online and a summary of their assessment results via email (within IRIS).

Assessment Results Letters (baseline and follow-ups) will report if the child’s CBCL scores fall within the clinical range for the Anxious/Depressed (CBCL 1½-5 & 6-18), Withdrawn/Depressed (CBCL/6-18), or Withdrawn (CBCL/1½-5) scales and will also include the parent’s depression, anxiety, and stress scores (DASS-21) as compared to the general population (ie, about the same, higher, or much higher than most people). Additionally, the child’s Externalizing CBCL scores will be reported as being the same, improved, worse, or resolved (within the normal range) as compared to baseline. Letters will emphasize to the participant that assessment results are not a diagnosis and do not replace medical care. Participants will be able to request a phone call from study staff to discuss assessment results.

### Statistical Analyses

Data will be analyzed by a statistician blinded to group assignment. The study design is a 2 (group: intervention vs control) × 3 (time: baseline, 5 months, 11 months) mixed factorial with repeated measures.

An intention-to-treat analysis will be performed using a full information maximum likelihood [31] mixed-effects regression framework. Specifically, for the primary outcome of the study we will create a hierarchical (“stacked”) data set and regress the CBCL externalizing scores on group (coded as Control=0 and Intervention=1), time (coded naturally as baseline=0, 5 months=5, etc) and the group × time interaction. Child age, sex, comorbidities, and medications have been identified a priori as variables to be added to the model. The critical test will be the group × time interaction. Based on the described coding, the parameter for this effect will be the estimated differential change on the CBCL externalizing score between the control and intervention groups per month. The overall effect will be this parameter estimate × 5. We anticipate using an unstructured covariance matrix for deriving the error term.

## Discussion

Research has identified significant gaps in the capacity to treat FASD [10,11]. Continued evaluation of services established to provide support for families affected by FASD will highlight where gaps in care persist and provide evidence to modify services to obtain best practices. Intervention programs that support children and their families by strengthening their home environment and support systems are critical to maximizing the children’s potential and modifying secondary effects [12]. Reviews in this area have recognized that the specific challenges experienced by families of children with FASD can vary significantly and, consequently, each individual requires a personalized management program [32]. The Strongest Families FASD Parenting Program, adapted from the evidence-based Strongest Families “Parenting the Active Child Program” [13-15], is designed to address many of these issues by providing personalized distance services to families with children exhibiting behavior problems.

### Conclusions

Recruitment began July 2014. This study will (1) evaluate the effectiveness of the Strongest Families FASD program in reducing behavior problems and improving caregiver stress; (2) determine the feasibility of the Strongest Families online FASD Parenting Program for caregivers; and (3) inform eHealth service delivery policy and potentially influence uptake of the Strongest Families-FASD Program. The ultimate goal is to improve timely access to evidence-based eHealth services for families living with FASD.

## Appendix

Multimedia Appendix 1 Grant Funding Agency (Canadian Institutes of Health Research) Peer Review.

## Abbreviations

CBCL: Child Behavior Checklist
CSQ-8: Client Satisfaction Questionnaire
DASS: Depression Anxiety and Stress Scale
FASD: fetal alcohol spectrum disorder
IRIS: Intelligent Research and Intervention Software
SFI: Strongest Families Institute

## References

[1] Sampson PD, Streissguth AP, Bookstein FL, Little RE, Clarren SK, Dehaene P, Hanson JW, Graham JM (1997). Incidence of fetal alcohol syndrome and prevalence of alcohol-related neurodevelopmental disorder. Teratology.

[2] Public Health Agency of Canada (2009). What Mothers Say: The Canadian Maternity Experiences Survey (MES) (2006-07).

[3] Jones KL, Smith DF (1973). Recognition of the fetal alcohol syndrome in early infancy. Lancet.

[4] Jones KL, Smith DW, Ulleland CN, Streissguth P (1973). Pattern of malformation in offspring of chronic alcoholic mothers. Lancet.

[5] Lemoine P, Harousseau H, Borteyru JP, Menuet JC (2003). Children of alcoholic parents--observed anomalies: discussion of 127 cases. Ther Drug Monit.

[6] Koren G, Nulman I, Chudley AE, Loocke C (2003). Fetal alcohol spectrum disorder. CMAJ.

[7] Health Canada Public Health Agency of Canada (2014). Health Canada.

[8] May PA, Baete A, Russo J, Elliott AJ, Blankenship J, Kalberg WO, Buckley D, Brooks M, Hasken J, Abdul-Rahman O, Adam MP, Robinson LK, Manning M, Hoyme HE (2014). Prevalence and characteristics of fetal alcohol spectrum disorders. Pediatrics.

[9] Stade B, Ali A, Bennett D, Campbell D, Johnston M, Lens C, Tran S, Koren G (2009). The burden of prenatal exposure to alcohol: Revised measurement of cost. Can J Clin Pharmacol.

[10] Caley LM, Winkelman T, Mariano K (2009). Problems expressed by caregivers of children with fetal alcohol spectrum disorder. Int J Nurs Terminol Classif.

[11] Streissguth A, Barr H, Bookstein F, Sampson P, Olson H (1999). The Long-Term Neurocognitive Consequences of Prenatal Alcohol Exposure: A 14-Year Study. Psychological Science.

[12] Kodituwakku PW (2010). A neurodevelopmental framework for the development of interventions for children with fetal alcohol spectrum disorders. Alcohol.

[13] McGrath PJ, Lingley-Pottie P, Thurston C, MacLean C, Cunningham C, Waschbusch DA, Watters C, Stewart S, Bagnell A, Santor D, Chaplin W (2011). Telephone-based mental health interventions for child disruptive behavior or anxiety disorders: randomized trials and overall analysis. J Am Acad Child Adolesc Psychiatry.

[14] Lingley-Pottie P, McGrath PJ, Andreou P (2013). Barriers to mental health care: perceived delivery system differences. ANS Adv Nurs Sci.

[15] Lingley-Pottie P, McGrath PJ (2007). Distance therapeutic alliance: The participant's experience. ANS Adv Nurs Sci.

[16] Wozney L, McGrath P, Newton A, Huguet A, Franklin M, Perri K, Leuschen K, Toombs E, Lingley-Pottie Patricia (2015). Usability, learnability and performance evaluation of Intelligent Research and Intervention Software: A delivery platform for eHealth interventions. Health Informatics J.

[17] (2015). CONSORT.

[18] Sweeney L, Erickson DB, Touyz SW, Nixon Reginald D V (2003). Parent-child interaction therapy: a comparison of standard and abbreviated treatments for oppositional defiant preschoolers. J Consult Clin Psychol.

[19] van de Wiel NM, Matthys W, Cohen-Kettenis P, van Engeland H (2003). Application of the utrecht coping power program and care as usual to children with disruptive behavior disorders in outpatient clinics: A comparative study of cost and course of treatment. Behavior Therapy.

[20] Achenbach T, Rescorla L (2000). Manual for the ASEBA preschool forms & profiles: an integrated system of multi-informant assessment.

[21] Achenbach T, Rescorla L (2001). Manual for the ASEBA school-age forms & profiles: an integrated system of multi-informant assessment.

[22] Lovibond S, Lovibond P (1995). Manual for the Depression Anxiety Stress Scales. 2nd edition.

[23] Henry JD, Crawford JR (2005). The short-form version of the Depression Anxiety Stress Scales (DASS-21): construct validity and normative data in a large non-clinical sample. Br J Clin Psychol.

[24] Morawska A, Sanders MR (2006). Self-administered behavioral family intervention for parents of toddlers: Part I. Efficacy. J Consult Clin Psychol.

[25] Larsen DL, Attkisson CC, Hargreaves WA, Nguyen TD (1979). Assessment of client/patient satisfaction: development of a general scale. Eval Program Plann.

[26] Atkison CC, Greenfield TK, Maruish ME (2004). The Client Satisfaction Questionnaire-8. The Use Of Psychological Testing For Treatment Planning And Outcomes third Edition, Volume 3.

[27] Bertrand J (2009). Interventions for children with fetal alcohol spectrum disorders (FASDs): overview of findings for five innovative research projects. Res Dev Disabil.

[28] Nash K, Rovet J, Greenbaum R, Fantus E, Nulman I, Koren G (2006). Identifying the behavioural phenotype in Fetal Alcohol Spectrum Disorder: sensitivity, specificity and screening potential. Arch Womens Ment Health.

[29] LaFrance Michael-Anne, McLachlan Kaitlyn, Nash Kelly, Andrew Gail, Loock Christine, Oberlander Tim F, Koren Gideon, Rasmussen Carmen (2014). Evaluation of the neurobehavioral screening tool in children with fetal alcohol spectrum disorders (FASD). J Popul Ther Clin Pharmacol.

[30] Green CR, Roane J, Hewitt A, Muhajarine N, Mushquash C, Sourander A, Lingley-Pottie P, McGrath P, Reynolds JN (2014). Frequent behavioural challenges in children with fetal alcohol spectrum disorder: A needs-based assessment reported by caregivers and clinicians. J Popul Ther Clin Pharmacol.

[31] Johnson DR, Young R (2011). Toward Best Practices in Analyzing Datasets with Missing Data: Comparisons and Recommendations. J Marriage Fam.

[32] Caley LM, Shipkey N, Winkelman T, Dunlap C, Rivera S (2006). Evidence-based review of nursing interventions to prevent secondary disabilities in fetal alcohol spectrum disorder. Pediatr Nurs.

