# Supplementary material for: Guided Internet-Based Parent Training for Challenging Behavior in Children With Fetal Alcohol Spectrum Disorder (Strongest Families FASD): Study Protocol for a Randomized Controlled Trial
Source: JMIR Res Protoc. 2015 Oct 13;4(4):e112. doi: 10.2196/resprot.4723 (PMC4704905; doi:10.2196/resprot.4723)
Supplement: Multimedia Appendix 1 [file resprot_v4i4e112_app1.pdf]

Canadian Institutes of Health Research / Instituts de recherche en santé du Canada

Notice of Decision / Avis de décision

Application Number/Numéro de la demande: 267391

Committee Code/Code du comité: PHE

Applicants/Candidats: Dr. Patrick John MCGRATH   Mrs. Heather CAUGHEY

With/Avec: Dr. N. MUHAJARINE   Mr. c. MUSHQUASH   Dr. J. REYNOLDS   Prof. A. SOURANDER

Institution paid/ Établissement payé: IWK Health Centre (Halifax)

Title/Titre: Parent training for challenging behaviour in children with Fetal Alcohol Spectrum Disorders (FASD): Strongest Families for FASD

Primary Inst./ Inst. principal: Human Development, Child and Youth Health

Other Related Inst./ Autres inst. connexes: Health Services and Policy Research

**Competition Outcome/Résultats du concours:**	Partnerships for Health System Improvement (PHSI)<br>November/Novembre 01, 2011
**Number in competition/Nbre de demandes dans le concours:**	61
**Number approved/Nbre de demandes approuvées:**	27

**Decision on your application/ Décision sur votre demande:**	Approved
**Average annual amount/ Montant annuel moyen:**	$133,286
**Equipment amount/ Montant pour les appareils:**	$0
**Term/Durée:**	3 **yrs/ans** 0 **months/mois**

Peer Review Committee Recommendation, for your information and use/
Recommandation du comité d'examen par les pairs, pour fins d'information et d'utilisation:

**Committee/Comité:**		Partnerships for Health System Improvement
**Application rank within the competition/ Rang de la demande dans ce concours:**		7
**Percent Rank Within the Competition/ Rang en pourcentage au sein du concours:**		11.48%
**Rating/ Cote:**	Potential Impact	4.26
	Scientific Merit	4.12
**Recommended average annual amount/ Montant annuel moyen recommandé:**		$133,286
**Recommended equipment amount/ Montant recommandé pour les appareils:**		$0

*** Applications receiving a score of less than 3.5 on any evaluation criteria will not be considered for Funding. / Les demandes qui ont reçu une note inférieure à 3.5 pour n'importe quel des critères d'évaluation ne sont pas admissibles.

This document is for information only. Official payment is stated on the CIHR Authorization for Funding.
Document à titre d'information seulement. Le paiement officiel est indiqué sur l'autorisation de financement des IRSC.

CIHR IRSC

Institute of Aboriginal Peoples' Health
Institute of Aging
Institute of Cancer Research
Institute of Circulatory and Respiratory Health
Institute of Gender and Health
Institute of Genetics
Institute of Health Services and Policy Research
Institute of Human Development and Child and Youth Health
Institute of Infection and Immunity
Institute of Musculoskeletal Health and Arthritis
Institute of Neurosciences, Mental Health and Addiction
Institute of Nutrition, Metabolism and Diabetes
Institute of Population and Public Health

Institut de la santé des Autochtones
Institut du vieillissement
Institut du cancer
Institut de la santé circulatoire et respiratoire
Institut de la santé des femmes et des hommes
Institut de génétique
Institut des services et des politiques de la santé
Institut du développement et de la santé des enfants et des adolescents
Institut des maladies infectieuses et immunitaires
Institut de l'appareil locomoteur et de l'arthrite
Institut des neurosciences, de la santé mentale et des toxicomanies
Institut de la nutrition, du métabolisme et du diabète
Institut de la santé publique et des populations

April 30, 2012

Dr. Patrick John MCGRATH
IWK Health Centre
5850/5980 University Ave
Halifax, Nova Scotia B3K 6R8

RE: Partnerships for Health System Improvement Grant: 2011-2012 Competition, "Parent training for challenging behaviour in children with Fetal Alcohol Spectrum Disorders (FASD): Strongest Families for FASD"

Dear Dr. MCGRATH:

On behalf of the Knowledge Translation Branch and its partners, we are pleased to inform you that your application referenced above has been approved for funding. Your Authorization for Funding will follow shortly in the mail.

The "Partnerships for Health System Improvement" Grant is led by the Knowledge Translation Branch. Please note that your grant is funded by CIHR Institute of Health Services and Policy Research and CIHR Institute of Human Development, Child and Youth Health. You are required to acknowledge CIHR and any relevant funding partners in communications and publications related to this project.

This CIHR grant is conditional upon the support from all partners outlined in your application. Please see attached annexe for the detailed commitments from the competition and project-specific partners. Please contact CIHR if there is a decrease in any of the commitments that have been pledged for this grant.

As CIHR does not notify co-applicants of the decision taken, we ask that you inform those individuals involved along with their research institutions (if different than your own) of the outcome of this application.

Please note that you will be required to submit a final performance report; a template and deadline will be provided.

Should you require additional information on the review process and funding, please contact Anne-Marie Poulin, Program Delivery Coordinator, at anne-marie.poulin@cihr-irsc.gc.ca or by phone at (613) 948-2899.

We wish you all the best in your research endeavours.

Sincerely,

Alison Bourgon
Acting Deputy Director, Knowledge Creation Programs Branch
Research Portfolio

cc: Financial Administrator, Institution Paid
Research Administrator, Institution Paid

298966-201111PHE-PHE-267391-10417-PHEA

CIHR IRSC

Institute of Aboriginal Peoples' Health
Institute of Aging
Institute of Cancer Research
Institute of Circulatory and Respiratory Health
Institute of Gender and Health
Institute of Genetics
Institute of Health Services and Policy Research
Institute of Human Development and Child and Youth Health
Institute of Infection and Immunity
Institute of Musculoskeletal Health and Arthritis
Institute of Neurosciences, Mental Health and Addiction
Institute of Nutrition, Metabolism and Diabetes
Institute of Population and Public Health

Institut de la santé des Autochtones
Institut du vieillissement
Institut du cancer
Institut de la santé circulatoire et respiratoire
Institut de la santé des femmes et des hommes
Institut de génétique
Institut des services et des politiques de la santé
Institut du développement et de la santé des enfants et des adolescents
Institut des maladies infectieuses et immunitaires
Institut de l'appareil locomoteur et de l'arthrite
Institut des neurosciences, de la santé mentale et des toxicomanies
Institut de la nutrition, du métabolisme et du diabète
Institut de la santé publique et des populations

April 30, 2012

Dr. Patrick John MCGRATH
IWK Health Centre
5850/5980 University Ave
Halifax, Nova Scotia B3K 6R8

Dear Dr. MCGRATH:

Congratulations on your success in the recent Canadian Institutes of Health Research funding competition. You should take great pride in your success, particularly in light of the very competitive nature of CIHR peer review.

As you know, peer review is the cornerstone of our research funding system. This process rests on the kind voluntarism of your colleagues at other institutions who generously gave their time to review your application.

The Canadian Institutes of Health Research is committed to building an innovative national health research enterprise. To this end we have undertaken the development of a renewed strategic plan for CIHR, our Health Research Roadmap which has required support from researchers, policy makers, the voluntary sector and the Canadian public. To meet CIHR goals, we must share our knowledge. That is why we encourage you to work with your institution to communicate to Canadians about the work you are doing. To simplify this process, we have developed guidelines on public communication which you can find on our website at http://www.cihr-irsc.gc.ca/e/30789.html.

Once again, congratulations and I wish you success in your research.

Yours sincerely,

Alain Beaudet, MD, Ph.D.
President

298968-201111PHE-PHE-267391-10417-CONGR

Application Number / Numéro de demande: 267391
Name of Applicant / Nom du chercheur: MCGRATH, Patrick John
Review Type / Type dévaluation: Committee Member 1/Membre de comité 1
Competition: 2011-11-01 Partnerships for Health System Improvement (PHSI)
Concours: 2011-11-01 Partenariats pour l'amélioration du système de santé (PASS)
Committee: Partnerships for Health System Improvement
Comité: Partenariats pour l'amélioration du système de santé

Potential Impact

Comments:

This is an important topic and this study has the potential to contribute to the body of knowledge to address a current and urgent health care problem and need. This project is a superb example of a much needed intervention for a highly neglected population. The research objectives were crafted in response to an important need for change as identified by the knowledge users. Good partnerships have been developed across multiple provinces. The knowledge exchange plan is well described and the ongoing relationship with decision makers well articulated. The study is important to those directly responsible for the coordination and provision of care for kids with FASD and their family members.

Application Number / Numéro de demande: 267391
Name of Applicant / Nom du chercheur: MCGRATH, Patrick John
Review Type / Type dévaluation: Committee Member 1/Membre de comité 1
Competition: 2011-11-01 Partnerships for Health System Improvement (PHSI)
Concours: 2011-11-01 Partenariats pour l'amélioration du système de santé (PASS)
Committee: Partnerships for Health System Improvement
Comité: Partenariats pour l'amélioration du système de santé

Scientific Merit

Comments:

Dr. Patrick McGrath (Principal Applicant) is a clinical psychologist, Professor of Psychology, Pediatrics and Psychiatry and Canada Research Chair at the Dalhousie University and VP Research at the IWK Health Centre. Dr. McGrath has substantial expertise in developing Internet-based interventions to meet the clinical needs of children and youth with mental health issues. His Strongest Families™ program for disruptive behaviour and anxiety was evaluated in several CIHR-funded RCTs, and is now available across Canada. He has published more than 20 randomized trials and has contributed substantially to the clinical trials literature. The study team is strong and has a diverse mix of skills and includes an interdisciplinary focus with complementary skills, policy and academic connections.

Scientific Merit
The proposal is very well written and the PI has made a convincing argument on the significance for this study. The strengths of the application include:
1)The intervention is grounded on a previously tested program (Strongest families) with kids with Oppositional Defiant Disorder (ODD) and Attention deficit hyperactively disorder (ADHD) on a different cohort (FASD population).
2)Specially trained coaches will be available to families and kids and will assist with customization and personalization of the intervention.
3)As there is diversity among families raising children with FASD, such as cultural and ethnic backgrounds, special care will be taken to develop culturally appropriate interventions for participants from First Nations, Inuit, and Metis communities. There is a very good process outlined to ensure that the Strongest families intervention is customized to a new population and the intervention is tested for its' feasibility. First stage includes surveying 100 families to make sure they are aware of all the problematic behaviors of children with FASD. Second stage includes working with 20 families to develop the text content for the intervention.
4)There is a very clear RCT trial proposed within this work and includes appropriate methods and analysis for its' evaluation.

The weaknesses of the study are the following:
1)It is not clear what the various components of the intervention are in the main proposal.
2)There is no mention of treatment fidelity checks, which is a potential flaw in the design of the project. If the intervention is so customized, how will the investigators make sure the intended dose was received and all of the components of the intervention administered?
3)There is a risk that the FASD kids and their families will not be amenable to this Strongest Families intervention as the parents are expected to make changes in their parenting styles. Given that some parents have their own mental issues, perhaps they too may need some type of treatment
4)It is not clear why an RCT trial is planned prior to a more feasibility/pilot testing trial as it is not clear if the adapted intervention will demonstrate changes in outcomes as outlined (the child's behavior and their social integration skills).

**Application Number / Numéro de demande:**	267391
**Name of Applicant / Nom du chercheur:**	MCGRATH, Patrick John
**Review Type / Type dévaluation:**	Committee Member 2/Membre de comité 2
**Competition:**	2011-11-01 Partnerships for Health System Improvement (PHSI)
**Concours:**	2011-11-01 Partenariats pour l'amélioration du système de santé (PASS)
**Committee:**	Partnerships for Health System Improvement
**Comité:**	Partenariats pour l'amélioration du système de santé

Potential Impact

Comments:

Brief Summary
This 3 year research proposal seeks to develop and evaluate a family-centered intervention the families (parents or caregivers) of children with fetal alcohol spectrum disorder (FASD) between the ages of 4 and 12 years. The intervention will be an Internet-based parent training program specifically adapted for the parents, families or caregivers of children with FASD who present significant behavioral challenges. The evaluation will include a randomized clinical trial (RCT) component whereby the feasibility and outcomes of those receiving the intervention will be compared to those of a control group not receiving the intervention.

Potential Benefit
Children with FASD suffer from neuro-behavioral deficits and usually present significant challenges to those responsible for their care and safety. The parents or guardians who care for these children, particularly those in rural and remote communities, often do not have access to the necessary support and detailed advice on how to mange with these symptoms. Strong letters of support for this initiative have been provided by such organizations as: The Children's Hospital of Eastern Ontario, the FASD Support Network of Saskatchewan, the Child Development Centre of the Hotel Dieu Hospital in Kingston, ON, the Manitoba FASD Centre, the FASD Diagnostic Clinic at St. Michael's Hospital, Toronto, ON, the Healthy Germantons Family Support Program in Sioux Lookout – Dryden, ON and the FASD Initiative of the Public Health Agency of Canada.

Strengths
Strength of the proposed research is that it builds on a successful program in place at the IWK Health Centre in Halifax, NS. The program is called Strongest Families. The Strongest Families program has already developed modules for attention deficit hyperactivity disorder (ADHD) and oppositional defiance disorder (ODD). Another strength of the Strongest Families program compared to care delivered in a clinical setting or office practice is its flexibility and convenience. This educational program for parents or guardians uses trained coaches to work with parents or caregivers—at times that are convenient for the family.

Potential Areas for Improvement
The scope of the proposed intervention is limited to FASD children aged 4 to 12 years; however FASD is a lifetime condition. The study team may wish to include a component which will help parents and caregivers deal with the challenges of their FASD children as they undergo the transition into adolescence and young adulthood.

Knowledge Transfer
The KT plan is aimed at diverse audiences and is very well described. KT activities will include linkage with current programs and initiatives of the Public Health Agency of Canada – such as the Community Acton Plan for Children and the Canada Prenatal Nutrition Program. Other linkages include a variety of FASD support networks and the Canadian Association of Pediatric Health Centers.

**Application Number / Numéro de demande:**	267391
**Name of Applicant / Nom du chercheur:**	MCGRATH, Patrick John
**Review Type / Type dévaluation:**	Committee Member 2/Membre de comité 2
**Competition:**	2011-11-01 Partnerships for Health System Improvement (PHSI)
**Concours:**	2011-11-01 Partenariats pour l'amélioration du système de santé (PASS)
**Committee:**	Partnerships for Health System Improvement
**Comité:**	Partenariats pour l'amélioration du système de santé

Scientific Merit

Comments:

Study Team
The study team is strong and diversified. It is led by a professor of psychology at Dalhousie University who also holds faculty appointments in pediatrics and psychiatry as well as the Canada Research Chair at Dalhousie. Other members of the study team have professional and research backgrounds in a number of areas pertaining to FASD. They include psychiatry, behavioral sciences, mental health, addictions, pharmacology, toxicology and epidemiology. The study team appears to have the experience and skills needed to successfully complete this research and development project.

Methods and Approach
The research plan consists of three consecutive studies. Study 1 will examine the behavioral and emotional problems of children diagnosed with FASD and their parents or guardians. Participants to be enrolled in the study will be identified using information obtained from the clinical records of organizations providing care to FASD patients and families. Participants will be selected so as to ensure at least 30% representation from First Nations, Métis and Inuit communities. Information will be gathered through telephone surveys (n=100) using a survey instrument developed by the principal investigator. Study 2 will use the findings of the telephone surveys to develop text content for illustrative teaching materials (30 video clips and 75 audio clips). These materials will be used in a progressive series of individualized interactive learning modules. A number of families (n=20) will be recruited to try out the modules and provide feedback as to their value and usability. Study 3 will consist of a randomized clinical trial (RCT) of 17 months duration. A total of 200 families with an FASD child will be recruited and assigned randomly to the intervention group or to the control group. The intervention group will receive the training program over the Internet via a personalized website – plus weekly verbal support from a trained "coach" over the telephone. The control group will not receive the intervention, but will be referred to available sources of information about the care of FASD children. The outcome variable to be analyzed in the RCT will be changes in the behavior of the FASD child assessed according to a scale called the Child Behavior Checklist (CBCL).

The use of a single variable to compare the different results achieved by the intervention group and the control group may not be sufficient to demonstrate conclusively that the educational intervention alone is capable of achieving significantly better outcomes in terms of child behavior. Overall, however, the methods and approach described in this study appear likely to yield findings and results that could be used in the improvement of other support programs for the parents or caaregivers of FASD children.

Potential Areas for Improvement
Since some parents of FASD children may still have alcohol related problems which impair their ability to provide good care, the study team may wish to consider adding an educational component to the study that would provide additional support and guidance to those who need it.

Budget
The total proposed budget for this 3 year study Is $567K of which $400K is being sought from CIHR. The balance of $167K will be provided in the form of cash and in-kind contributions from study partners such as the Saskatchewan Population Health and Research Unit, NeuroDevNet (a network of centres of excellence in developmental neuroscience, and the NS Center for Research in Family Health. Major

Scientific Merit

Comments:

personnel expenditures include a research associate (0.5 FTE), a research, coordinator ((0.5 FTE), a research assistant, (0.5 FTE), a clinical manager (0.1 FTE) and a “coach” (1.0 FTE) to work with the study participants in years 2 and 3. The proposed expenditure items are consistent with the work plan and appear reasonable and appropriate.

Review Type/Type dévaluation: SO Notes /Notes de l'agent scientifique
Name of Applicant/Nom du chercheur: MCGRATH, Patrick John
Application No./Numéro de demande: 267391
Agency/Agence: CIHR/IRSC
Competition/Concours: 2011-11-01 Partnerships for Health System Improvement (PHSI)/Partenariats pour l'amélioration du système de santé (PASS)
Committee/Comité: Partnerships for Health System Improvement/Partenariats pour l'amélioration du système de santé
Title/Titre: Parent training for challenging behaviour in children with Fetal Alcohol Spectrum Disorders (FASD): Strongest Families for FASD

Assessment/Évaluation:

This program offers a systematic approach to adapt and adopt a previously successful intervention program in a new target population (FASB). There is a convincing argument about the need for the program, an aim to personalize the program and adapt and consider cultural considerations. There is an opportunity to better assess the intervention fidelity. They may need to specifically address (not treat but consider) parental mental health needs? The researchers may also wish to consider issues that will arise at the upper limit of the program's eligibility criteria as clients transition out of the program. A strong KT plan is described including translation across Canada and internationally.
